# Identification of immunological patterns characterizing immune-related psoriasis reactions in oncological patients in therapy with anti-PD-1 checkpoint inhibitors

**DOI:** 10.3389/fimmu.2024.1346687

**Published:** 2024-03-01

**Authors:** Martina Morelli, Maria Luigia Carbone, Giovanni Luca Scaglione, Claudia Scarponi, Valentina Di Francesco, Sabatino Pallotta, Federica De Galitiis, Siavash Rahimi, Stefania Madonna, Cristina Maria Failla, Cristina Albanesi

**Affiliations:** ^1^ Experimental Immunology Laboratory, Istituto Dermopatico dell’Immacolata (IDI-IRCCS), Rome, Italy; ^2^ Department of Dermatology, Istituto Dermopatico dell’Immacolata (IDI-IRCCS), Rome, Italy; ^3^ Department of Oncology, Istituto Dermopatico dell’Immacolata (IDI-IRCCS), Rome, Italy; ^4^ Anatomical Pathology Unit, Istituto Dermopatico dell’Immacolata (IDI-IRCCS), Rome, Italy

**Keywords:** psoriasis, melanoma, anti-PD-1 therapy, immune-related cutaneous adverse event (ircAE), paradoxical skin reactions, adaptive immunity, innate immunity

## Abstract

**Introduction:**

Immunotherapy with biologics targeting programmed cell death protein-1 (PD-1) is highly effective in the treatment of various malignancies. Nevertheless, it is frequently responsible for unexpected cutaneous manifestations, including psoriasis-like dermatitis. The pathogenesis of anti-PD-1-induced psoriasis has yet to be clarified, even though it is plausible that some innate and adaptive immunity processes are in common with canonical psoriasis. The genetic predisposition to psoriasis of patients could also be a contributing factor. Here, we investigated the immunological and genetic profiles of two patients with metastatic melanoma and one patient affected by lung cancer, who developed severe psoriasis after receiving anti-PD-1 nivolumab therapy.

**Methods:**

The immune patterns of the three patients were compared with those detectable in classical, chronic plaque-type psoriasis or paradoxical psoriasis induced by anti-TNF-α therapy, mostly sustained by adaptive and innate immunity processes, respectively. Therefore, immunohistochemistry and mRNA analyses of innate and adaptive immunity molecules were conducted on skin biopsy of patients. Genetic analysis of polymorphisms predisposing to psoriasis was carried out by NGS technology.

**Results:**

We found that anti-PD-1-induced psoriasis showed immunological features similar to chronic psoriasis, characterized by the presence of cellular players of adaptive immunity, with abundant CD3^+^, CD8^+^ T cells and CD11c^+^ dendritic cells infiltrating skin lesions, and producing IL-23, IL-6, TNF-α, IFN-γ and IL-17. On the contrary, a lower number of innate immunity cells (BDCA2^+^ plasmacytoid dendritic cells, CD15^+^ neutrophils, CD117^+^ mast cells) and reduced IFN-α/β, lymphotoxin (LT)-α/β, were observed in anti-PD-1-induced psoriasis lesions, as compared with anti-TNF-α-induced paradoxical psoriasis. Importantly, the disintegrin and metalloprotease domain containing thrombospondin type 1 motif-like 5 (ADAMTSL5) psoriasis autoantigen was significantly upregulated in psoriasis lesions of anti-PD-1-treated patients, at levels comparable with chronic plaque-type psoriasis. Finally, NGS analysis revealed that all patients carried several allelic variants in psoriasis susceptibility genes, such as *HLA-C*, *ERAP1* and other genes of the major psoriasis susceptibility *PSORS1* locus.

**Discussion:**

Our study showed that adaptive immunity predominates over innate immunity in anti-PD-1-induced psoriasis lesions, consistently with the local ADAMTSL5 overexpression. The presence of numerous SNPs in psoriasis susceptibility genes of the three patients also suggested their strong predisposition to the disease.

## 1 Introduction

Immune checkpoint inhibitors (ICIs) are increasingly used as first-line therapy in various malignancies, including melanoma and lung cancer (1, 2). In these conditions, treatment with monoclonal antibodies against immune checkpoint molecules, such as programmed cell death protein-1 receptor (PD-1) and/or cytotoxic T lymphocyte-associated protein 4 (CTLA-4), can result in enhanced cytotoxic activation of tumor antigen-specific T cells and in eradication of tumor lesions. However, immune activation by ICIs may lead to immune-mediated adverse events, among which cutaneous reactions are the most common (approximately 40%) (3, 4). Among immune-related cutaneous adverse events (ircAE), maculopapular rashes, lichenoid eruptions, and vitiligo, as well as less common inflammatory and autoimmune manifestations, such as hidradenitis suppurativa (HS) and *de novo* or worsening of pre-existing psoriasis, have been described (3–5). Most ircAE are of low-grade and are treated with corticosteroids, a therapy that seems not to interfere with the anti-tumor immune responses activated by ICIs. Severe ircAEs have been observed in the 1-2% of patients and may require additional topical or systemic agents, including antihistamine compounds and biologics. In the case of ircAEs, dose changes were made in up to 21% of patients, whereas therapy discontinuation was necessary only in about 8% of patients (4).

Pathogenically, ircAEs induced by anti-PD-1 therapies result from breaking of peripheral T cell tolerance and unleashing of immune and inflammatory responses, likely dependent on CD8^+^ T cells and IL-6 enhanced production (6–8). For instance, PD-1 signal blockade-induced psoriasis-like dermatitis is characterized by a prominent epidermal infiltration of CD8^+^ T cells and overexpression of IL-6, IL-23 and IL-17A cytokines, as demonstrated in a murine model of psoriasis induced by imiquimod (IMQ), carried out with genetically modified mice lacking PD-1 in CD8^+^ T cells (8).

An aberrant migration and accumulation of CD8^+^ T lymphocytes into the epidermis has also been shown in classical psoriasis, especially during the establishment of adaptive immune responses (9). In stable plaques, intraepidermal CD8^+^ T cells display highly pathogenic features, as they abundantly produce cytokines, such as IL-17A and IFN-γ, which in turn dictate specific and inflammatory gene signatures in keratinocytes and in other resident skin cells (10). The dialogue between CD8^+^ T lymphocytes and keratinocytes, as well as with skin cells of adaptive immunity, such myeloid dendritic cells (mDC), is strictly depends on the recognition of peptide (auto)antigens presented by MHC class I molecules, such as *HLA-Cw6*, which is the strongest psoriasis susceptibility allele (11). Among them, the disintegrin and metalloprotease domain containing thrombospondin type 1 motif-like 5 (ADAMTSL5), a protein modulating microfibril functions, has been identified as autoantigen presented by melanocytes and keratinocytes in an HLA-Cw6-restricted fashion (12), and localized throughout the psoriatic epidermis (13, 14).

Systemic administration of anti-TNF-α biologics can be responsible for unexpected paradoxical psoriasiform reactions in patients treated for immune-mediated inflammatory conditions, such as HS, rheumatoid arthritis, and inflammatory bowel disease (15, 16). Our previous studies on the characterization of anti-TNF-α-induced paradoxical psoriasis reactions revealed an overactivation of innate immunity in the skin lesions of HS patients, also due to a strong predisposition of HS patients to develop immune responses against innate stimuli, and the presence of an immunological infiltrate mainly represented by BDCA2^+^ plasmacytoid dendritic cells (pDCs), CD15^+^ neutrophils, c-kit/CD117^+^ mast cells, CD68^+^ macrophages and monocytes (17). A local overproduction of the type I IFNs, IFN-β and IFN-α2a, concomitantly to other innate immunity molecules, such as lymphotoxin (LT)-α and LT-β, was also detected in paradoxical psoriatic skin of patients treated with anti-TNF-α biologics (17).

To date, some of the pathogenic mechanisms underlying immune-related psoriasis reactions in patients treated with anti-PD-1 have been described, even though the specific patterns of innate and adaptive immunity prevailing in the skin lesions are still to be elucidated. The influence of genetic predisposition of patients to psoriasis is also unproven.

In this study, we investigated skin immunological patterns of two patients with metastatic melanoma and one patient affected by lung cancer, who developed severe psoriasis after receiving anti-PD-1 nivolumab therapy. The immune patterns characterizing psoriasis lesions of patients were compared with those detectable in classical, stable plaque-type psoriasis or paradoxical psoriasis induced by anti-TNF-α therapy, mostly sustained by adaptive and innate immunity processes, respectively. The expression of the psoriasis autoantigen ADAMTSL5 and the genetic susceptibility to psoriasis of the three patients were also studied.

## 2 Materials and methods

### 2.1 Patients and samples

Two patients with metastatic melanoma, stage IV (AJCC, version 8) (18), and one patient affected by metastatic lung cancer, stage IVB (19), and developing psoriasis after receiving nivolumab (240 mg every 2 weeks) were included in the study.

Six patients affected by classical plaque-type psoriasis (Psoriasis area and severity index, PASI: 8-21 range), and three patients with severe HS (Hurley III, Sartorius score: 41.5-61.5 range) showing paradoxical psoriasis after treatment with adalimumab (40 mg, weekly) were also enrolled for the study. Clinical data, as well as skin biopsies and blood, were collected from patients with the permission of the IDI-IRCCS Local Ethics Committee (Prot. CE 475/2016). The participants provided their written informed consent to participate in this study.

For oncological patients, the investigator-determined objective response was assessed radiologically with computed tomography scans approximately every 12 weeks after treatment initiation. Tumor response was classified according to the immune response evaluation criteria in solid tumors (iRECIST 1.1) (20), and therapy efficacy evaluation was based on best overall response determined as best time-point response according to iRECIST. Eight-mm skin biopsies were taken from psoriasiform lesions arising in enrolled patients. For patient 2, biopsy specimens from primary and melanoma skin metastasis, as well as from vitiligo lesions were collected from the archives of the Anatomical Pathology Unit of IDI-IRCCS. Skin biopsies were also taken from normal-appearing, non-lesional skin of psoriatic patients. Biopsies were divided into two parts for immunohistochemistry and RNA isolation. A 2-ml sample of peripheral blood was used to extract DNA.

### 2.2 Immunohistochemistry

Skin samples from healthy donors, immune-related psoriasis induced by anti-PD-1, chronic plaque-type psoriasis and anti-TNF-α-induced paradoxical psoriasis. were fixed in 10% formalin and embedded in paraffin. Five-μm sections were dewaxed and rehydrated and stained with hematoxylin and eosin (H&E) or processed for immunohistochemistry. In this case, endogenous peroxidase was quenched by 3% H_2_O_2_ treatment and then antigen retrieval was achieved by treating sections with citrate buffer pH 6.0 or Tris-EDTA buffer pH 7.8 (both from UCS Diagnostic, Rome, Italy), depending on the primary antibodies (Abs). After blocking nonspecific binding sites with a blocking solution (Dako, Glostruk, Denmark), sections were incubated with the primary Abs. The latter were as follows: anti-CD3 (#A0452, Dako, 1:100 dilution), anti-CD8 and anti-IFN-α2A (#AB217344, 1:75 dilution and, #AB198914, 1:75 dilution, respectively; both were from Abcam, Cambridge, UK), anti-CD11c and anti-CD117 (#MON3371, 1:50 dilution and #MONX10234, 1:100 dilution, respectively; both Abs were purchased from Monosan, Uden, Netherlands), anti-BDCA2 (DDX0043-TDS, Dendritics, Lyon, France, 1:30 dilution), anti-CD15 (#347420, BD Biosciences, Milan, Italy, 1:30 dilution), anti-IL-17A (#AF-317-NA, R&D Systems, Abingdon, UK, 1:30 dilution) and anti-ADAMTSL5 (#NBP1-93438, Novus Biologicals, Centennial, USA, 1:50 dilution). Immunoreactivity was visualized with peroxidase reaction using 3-amino-9-ethylcarbazole (AEC) or 3,3’-diaminobenzidine (DAB) in H_2_O_2_, and specimen counterstained with hematoxylin. As a negative control, the primary Abs were omitted or replaced with an irrelevant isotype-matched Ab. Positivity was evaluated in five adjacent fields at a 200X magnification. Cells infiltrating dermis and epidermis were also counted in five adjacent fields for each skin specimen.

### 2.3 Real-time PCR analysis

Total RNA was extracted from skin biopsies using RecoverAll Total Nucleic Acid Isolation (Life Technologies). mRNA was reverse transcribed into cDNA by using SuperScript IV VILO master mix (Invitrogen) and analyzed by QuantStudio5 real-time PCR System (Thermo-Fisher Scientific, Waltham, MA, USA) using SYBRGreen or Taqman PCR reagents. The primer sets were as follows: IFN-β, 5’CAGCAATTTTCAGTGTCAGAAGC3’/5’TCATCCTGTCCTTGAGGCAGT3’; LT-α, 5’CTACCGCCCAGCAGTGTC3’/5’GGTGGTGTCATGGGGAGA3’; LT-β, 5’GGCGGTGCCTATCACTGT3’/5’GAAACCCCAGTCCTTGCTG3’; TNF-α, 5’CTCTTCTGCCTGCTGCACTTTG3’/5’ATGGGCTACAGGCTTGTCACTC3’; IL-6, 5’GGCACTGGCAGAAAACAACC3’/5’CACCAGGCAAGTCTCCTCAT3’; IL-23, 5’GACAACAGTCAGTTCTGCTTGC3’/5’GAGAAGGCTCCCCTGTGAAA3’; β2M, 5’GATGAGTATGCCTGCCGTGTG3’/5’CAATCCAAATGCGGCATCT3’. IL-17A, IL-22 and IFN-γ genes were analyzed by the TaqMan gene expression assay (assay ID: Hs00174383_m1, Hs00220924_m1 and Hs00174143_m1, respectively). mRNA levels were normalized to β2M mRNA expression. The values obtained from triplicate experiments were averaged, and data presented as mean 2^−ΔΔCT ± SD.

### 2.4 SNP analysis

DNA was extracted from blood using the QIAcube^®^system (Qiagen, Hilden, Germany), and 10 ng were used for high-throughput sequencing by NGS technology. SNPs were selected based on an extensive review of articles on the association between psoriasis and SNPs or response to biological therapeutics (21–27). The customized designed SNP panel permitted to identify 417 genetic variants together with additional SNPs located in proximity of the investigated genomic regions. The SNP panel was analyzed by targeted sequencing, using Ion AmpliSeq™ Library kit Plus (Thermo Fisher Scientific) and the Ion GeneStudio™ S5 Plus platform (Thermo Fisher Scientific, Massachusetts, USA). Sequencing data were processed with the Ion Torrent Suite software v.5.10. Positive calls were selected applying a read depth>30X and allelic frequency >0.3. Reads were aligned to human genome sequence (build GRCh37/human genome 19). Variants were collected using Variant Caller. Variants’ annotations were finally verified using ANNOVAR.

### 2.5 Statistics

The significance of differences in the numbers of immunoreactive cells in skin biopsies was calculated using the unpaired Student’s *t*-test and values are expressed as the median + interquartile range. Unpaired Student’s *t*-test was also used to compare differences in mRNA content in skin biopsies of patients. All statistical analysis were conducted using Prism v.10.1.0 (GraphPad Software, Boston, MA, USA) and statistical significance was assumed at a *p* value of 0.05 or less.

## 3 Results

### 3.1 Clinical characterization of immune-related psoriasis reactions in patients undergone anti-PD-1 therapy

We studied two patients with metastatic melanoma and one patient affected by lung cancer, who developed cutaneous reactions after receiving anti-PD-1 immunotherapy.

**Figure 1:**
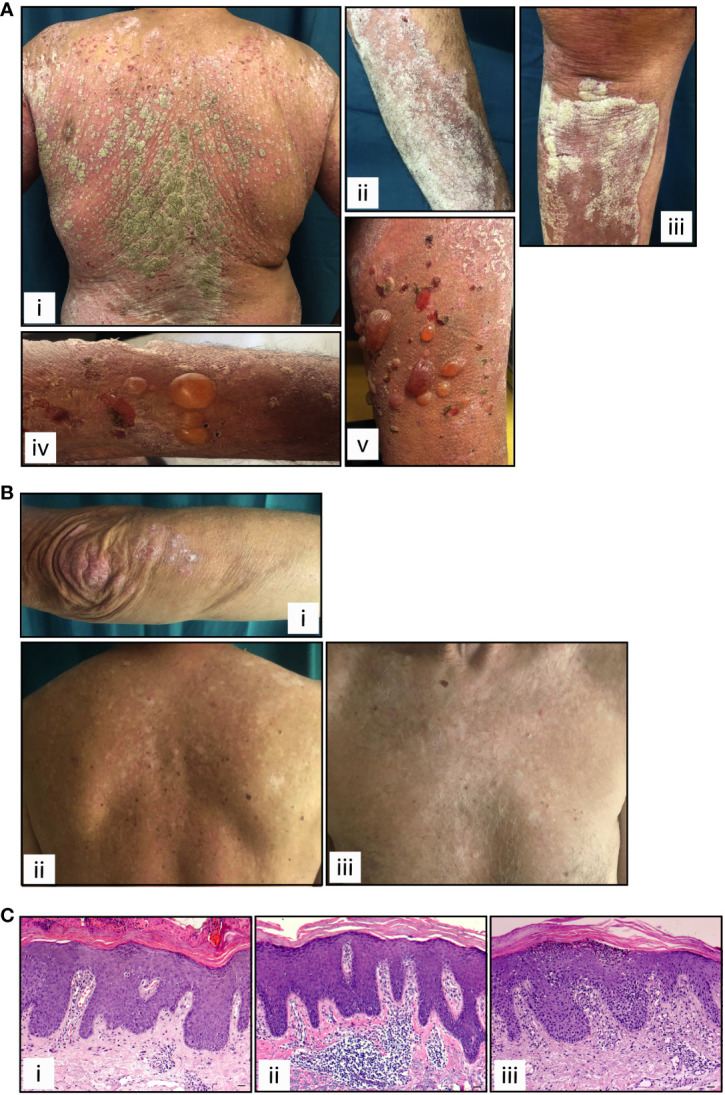
Clinical and histological presentation of psoriasis induced by anti-PD-1 therapy. Cutaneous manifestations of patients 1 and 2 affected by lung cancer and melanoma, respectively, presenting psoriasis reaction after receiving anti-PD-1 treatment. **(A)** Patient 1, panels i-iii show paradoxical erythemato-squamous plaques localized on the trunk, arms and lower limbs. Patient 1 also developed generalized bullous pemphigoid, arising after treatment with nivolumab (panels iv-v). **(B)** Patient 2 shows squamous plaques on the elbows and upper limbs (i) and on the trunk (ii), concomitantly with vitiligo (ii and iii). **(C)** H&E staining for the corresponding histology specimens of patients 1 (i), patient 2 (ii) and patient 3 (iii) was also performed. Scale bars, 20 μm.

Patient 1, a 70-year-old Caucasian man, was affected by metastatic lung cancer and, considering the best overall response, he responded positively to nivolumab therapy with an immune complete response (iCR, **Table 1**). He reported a personal history of psoriasis, and after 2-week treatment with anti-PD-1, showed a re-occurrence of the disease (PASI 21) (**Figure 1A**, panels i-iii). For psoriasis condition, patient 1 received therapy with systemic dexamethasone and topical clobetasol, which however resulted unsuccessful. Patient 1 also developed bullous pemphigoid, arising after 100-week treatment with nivolumab (**Figure 1A**, panels iv-v). Patient 2, a 67-year-old Caucasian man, was affected by metastatic melanoma and, after treatment with anti-PD-1 immunotherapy he achieved an iCR as the best response (**Table 1**). After 20 weeks of ICI therapy, he showed vitiligo, and after 52 weeks of treatment he concomitantly developed plaque-type psoriasis on the legs, elbows, and trunk (PASI 8) (**Figure 1B**). Patient 2 was not treated for psoriasis condition, neither systemically nor topically. Patient 2 reported previous psoriasis. Anti-PD-1 therapy was not discontinued in patient 1 and 2, even after ircAE manifestation (**Table 1**). Finally, a 62-year-old Caucasian woman, patient 3, with metastatic melanoma treated with anti-PD-1 and with an immune unconfirmed progressive disease (iUPD) as the best response, after 4 weeks of immunotherapy developed psoriasis (PASI 15) and discontinued ICI treatment for this ircAE. For psoriasis condition, she was successfully treated with acitretin and with topical corticosteroids. Patient 3 never restarted anti-PD-1 therapy for the re-occurrence of psoriasis condition. Interestingly, none of patients had a positive family history for psoriasis (**Table 1**), neither showed psoriasis reactions before ICI treatment. Histological examination of the psoriasis lesions of all the patients showed epidermal hyperplasia with parakeratosis, papillary vessel ectasia and perivascular infiltrate compatible with a psoriasiform dermatitis (**Figure 1C**). A neutrophilic infiltrate was present in corneal abscesses (**Figures 1C**).

**Table 1:** Characteristics and treatment outcomes of enrolled patients.

Characteristics	Patient 1	Patient 2	Patient 3
Sex	male	male	female
Age^a^	70	67	62
BMI^b^	31.96	24.76	22.86
Disease	lung cancer	melanoma	melanoma
Stage^c^	IVB	IV	IV
Checkpoint inhibitor^c^	nivo	nivo	nivo
Best Response^d^	iCR	iCR	iUPD
ircAEs (week)^e^	Psoriasis (2), Bullous Pemphigoid (100)	Psoriasis (52), Vitiligo (20)	Psoriasis (4)
PASI (ircAE)	21	8	15
ICI discontinuation for ircAEs	no	no	yes
Previous psoriasis	yes	yes	no
Psoriasis family history	no	no	no

a Age, years.

b BMI, (kg/m^2^)

c Staging before starting immunotherapy: nivo, nivolumab.

d Best Response according to the iRECIST criteria, at the last observation: iCR, immune complete response.

iUPD, immune unconfirmed progressive disease.

e Weeks of treatment before the onset of immune-related cutaneous adverse events (ircAEs); PASI, psoriasis area severity index.

### 3.2 Adaptive immunity predominates over innate immune responses in psoriasis lesions induced by anti-PD-1 therapy

**Figure 2:**
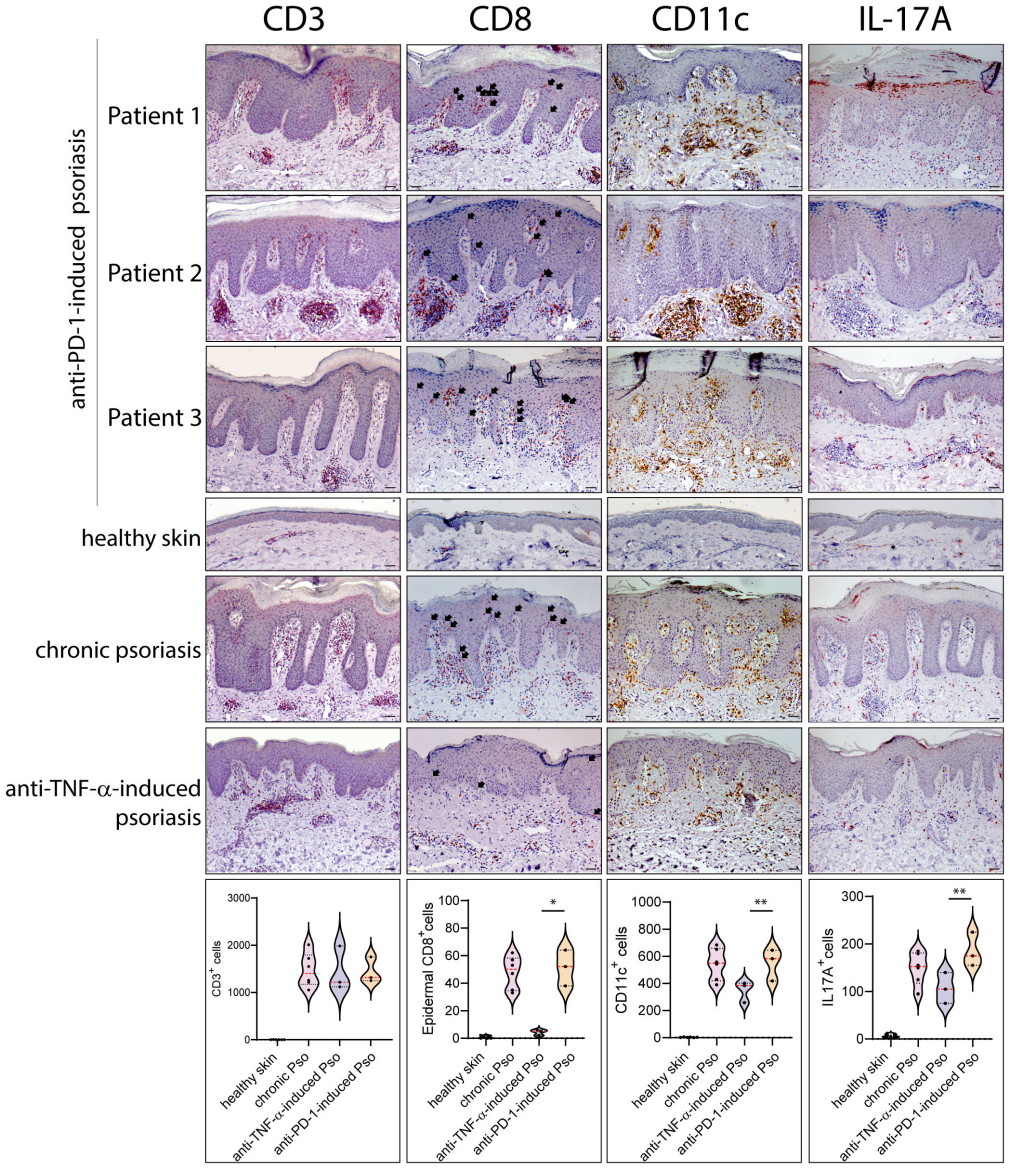
Paradoxical psoriasis induced by PD-1 blockade show a prominent infiltrate of CD3^+^, CD8^+^ T cells, CD11c^+^ DC and IL-17^+^ cells, at levels and patterns of distribution similar to chronic psoriasis. Leukocyte subpopulations were characterized by immunohistochemistry in paradoxical psoriasis lesions induced by anti-PD-1 (Patient 1, 2 and 3), and compared to those present in chronic plaque-type psoriasis (*n*=6 patients) and in paradoxical psoriasis induced by anti-TNF-α therapy (*n*=3). The distribution of numerical data relative to cell immunoreactivity for CD3 (red staining), CD8 (red), CD11C (brown), and IL-17A (red staining) in the three types of psoriasis reactions, are represented in the violin plots. Immunohistochemistry analysis of anti-PD-1 psoriasis skin reactions obtained from patient 1, patient 2 and 3 shows similar numbers of CD3^+^ cells and higher number of epidermal CD8^+^, dermal CD11C^+^, and IL-17A^+^ cells, when compared with paradoxical psoriasis induced by anti-TNF-α. Chronic psoriasis and anti-PD-1-induced psoriasis showed similar values in immunoreactive cells. No immunoreactivities were observed in skin samples from healthy donors (*n*=6). Arrows indicate CD8^+^ T cells localized within epidermis. Slides were analyzed by two pathologists with experience in dermatology. Positive cells were counted in five adjacent fields at a total magnification of ×200. For chronic or anti-TNF-α-induced psoriasis, one representative set of staining is shown. For each patient, one out of three representative stainings is shown. *p < 0.05, **p < 0.01 versus anti-TNF-α-induced psoriasis. Scale bars, 40 μm.

**Figure 3:**
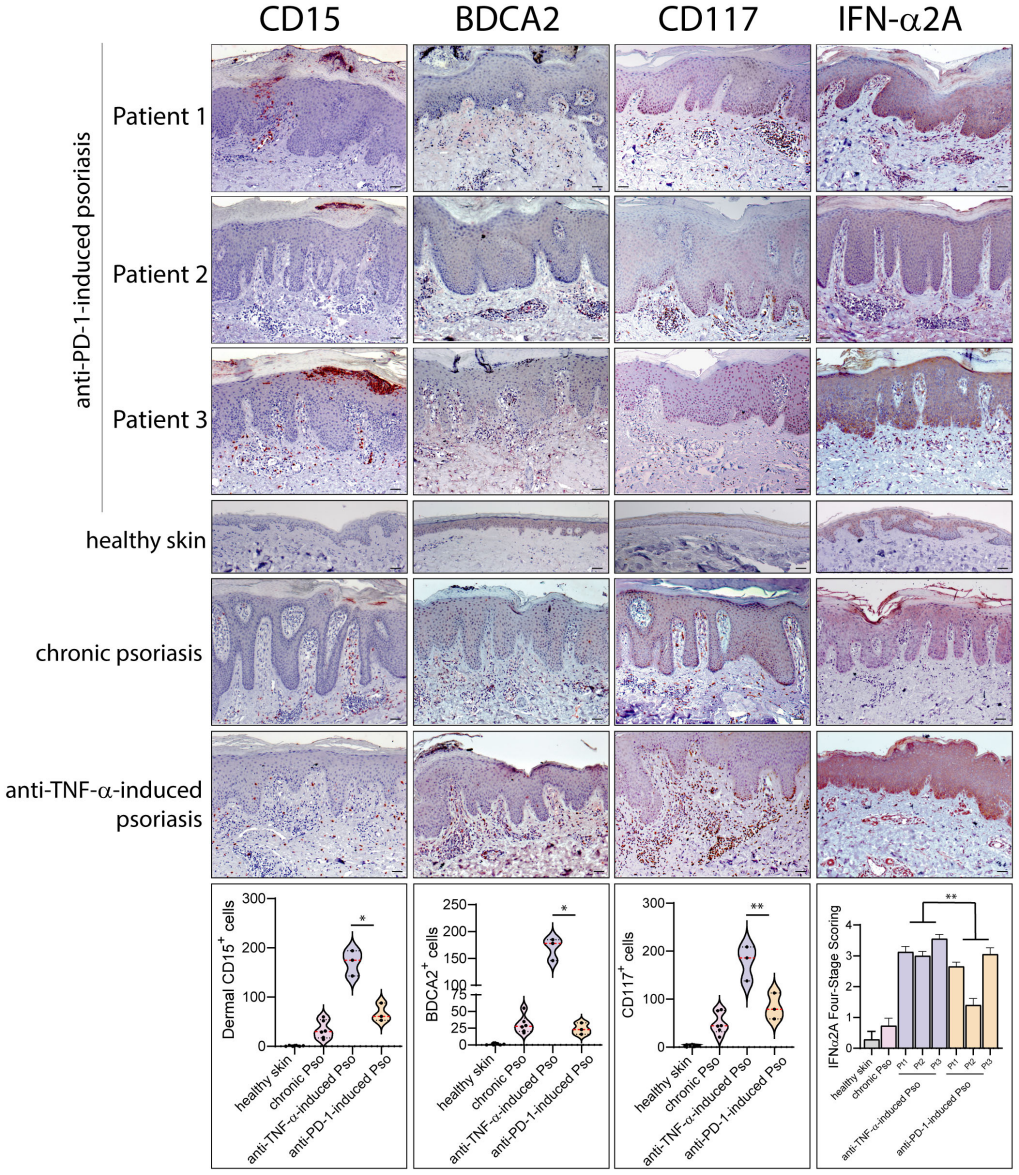
Innate immunity patterns are lacking in anti-PD-1-induced psoriasis. Innate immunity cell subpopulations were characterized by immunohistochemistry in paradoxical psoriasis lesions induced by anti-PD-1 (Patient 1, 2 and 3), and compared to those present in chronic plaque-type psoriasis (*n*=6 patients) and in paradoxical psoriasis induced by anti-TNF-α therapy (*n*=3). The distribution of numerical data relative to cell immunoreactivity for CD15 (red staining), BDCA2 (brown) and c-kit/CD117 (red staining) in the three types of psoriasis reactions are represented in the violin plots. Graph shows the mean ± SD of semiquantitative, four-stage scoring, ranging from negative immunoreactivity (0) to strong immunoreactivity (4+) of IFN-α2A staining (red). The infiltrate of dermal CD15^+^ neutrophils BDCA2^+^ pDC, and c-kit/CD117^+^ mast cells, was less abundant in immune-related psoriasis induced by anti-PD-1 as compared to paradoxical psoriasis induced by anti-TNF-α. IFN-α2A also was less abundant in psoriasiform reactions of anti-PD-1-treated patients, as compared to paradoxical psoriasis induced by anti-TNF-α. Similar immunoreactivity values were observed in anti-PD-1-induced and chronic psoriasis. No significant immunoreactivities were observed in skin samples from healthy donors (*n*=6). Slides were analyzed by two pathologists with experience in dermatology. Positive cells were counted in five adjacent fields at a total magnification of ×200. For chronic or anti-TNF-α-induced psoriasis, one representative set of reactions is shown. For each patient, one out of three representative staining is shown. *p < 0.05, **p < 0.01 versus anti-TNF-α-induced psoriasis. Scale bars, 40 μm.

Leukocyte subpopulations were characterized by immunohistochemistry in immune-related psoriasis induced by anti-PD-1 and compared to those present in chronic plaque-type psoriasis (*n*=6 patients) and in paradoxical skin lesions of HS patients undergone anti-TNF-α therapy (*n*=3) (**Figures 2**, **3**). Immune-related psoriasis induced by PD-1 blockade exhibited immunological aspects of chronic inflammation, as skin lesions of all patients showed a prominent infiltrate of CD3^+^, CD8^+^ T cells and CD11c^+^ DC, at levels and patterns of distribution similar to stable psoriasis (**Figure 2**). In fact, CD8^+^ T lymphocytes and CD11c^+^ DC massively localized in the epidermis and in the papillary dermis, respectively, together with CD15^+^ neutrophils accumulating in the epidermis to form focal subcorneal aggregates (micro abscesses of Munro) (**Figure 2**). In contrast, anti-PD-1-induced psoriasis did not show the immunological signs of paradoxical psoriasis induced by TNF-α blockade or acute psoriasis, characterized by an overactivation of innate immunity pathways and prominent infiltration of innate immunity cells. CD15^+^ neutrophils, BDCA^+^ pDC, and c-kit/CD117^+^ mast cells, poorly infiltrated the mid and interpapillary dermis of skin lesions of the three patients, differently to what observed in anti-TNF-α-induced psoriasis lesions (**Figure 3**).

The quantification of immunoreactivity for markers for different leukocyte subpopulations showed that chronic psoriasis and anti-PD-1-induced lesions were characterized by a higher number of epidermal CD8^+^ T cells, dermal CD11c^+^ DCs and IL-17A^+^ cells than psoriasiform lesions induced by TNF-α blockade (~11.4-, 1.4- and 1.3-fold increase, respectively) (**Figure 2**). Conversely, the infiltrate of dermal CD15^+^ neutrophils BDCA^+^ pDC, and c-kit/CD117^+^ mast cells, was less abundant (~3.8-, 6.3-, and 2.8-fold decrease, respectively) (**Figure 3**). Psoriasiform reactions of anti-PD-1-treated patients were weakly immunoreactive for the type I IFN-α2A, at lower levels than paradoxical psoriasis induced by anti-TNF-α (~1.6-fold decrease) (**Figure 3**).

**Figure 4:**
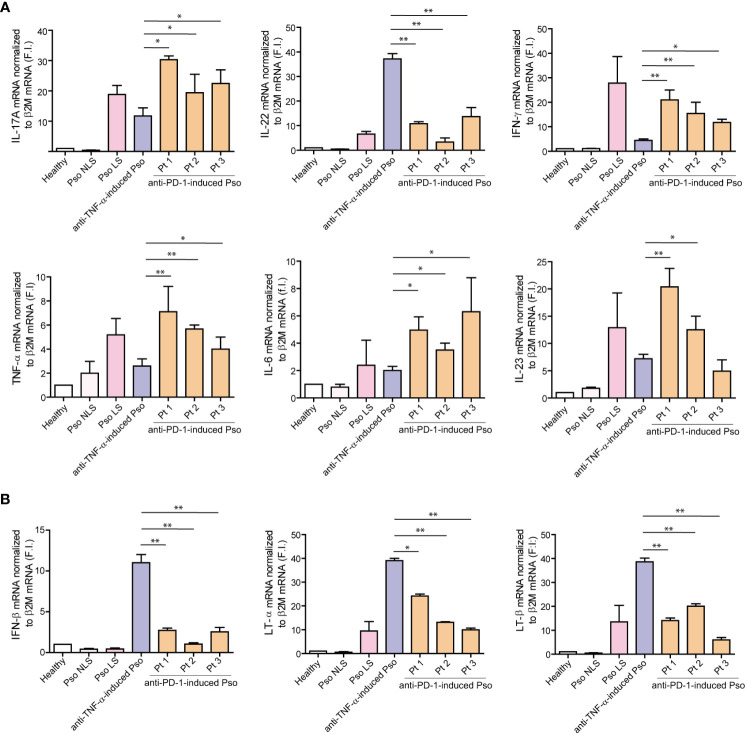
Inflammatory cytokines typical of chronic psoriasis are overexpressed in immune-related psoriasis reactions to anti-PD-1. **(A)** mRNA expression of IL-17A, IL-22, IFN-γ, TNF-α, IL-6 and IL-23 was analyzed by real-time PCR in psoriasis lesions induced by anti-PD-1 (Pt 1, Pt2 and Pt3), and compared to those present in skin biopsies from lesional (Pso LS) and non-lesional (Pso NLS) skin of psoriatic patients (*n*=6) and in paradoxical psoriasis induced by anti-TNF-α (*n*=3). Healthy skin from healthy donors (Healthy, n=6) was also analyzed. Levels of all mRNAs were significantly higher in the skin of the three patients, as compared to psoriasis-like reactions to anti-TNF-α, and similar to those detected in chronic psoriasis plaques. **(B)** Real-time PCR analysis of selected innate immunity molecules showed that IFN-β, LT-α and LT-β mRNAs are less expressed in psoriasis lesions induced by anti-PD-1, as compared to psoriasis reactions to anti-TNF-α. mRNA values were normalized to β2M mRNA. All data shown are the mean of three different experiments ± SD. Statistical significance was assessed by paired Student’s *t* test, *p ≤ 0.05, **p ≤ 0.01.

Consistently with a prevalence of adaptive immunity over innate immune responses in psoriasis induced by anti-PD-1 therapy, we found that the mRNA expression levels of psoriasis-related cytokines, such as IL-17A, IL-23, IFN-γ and IL-22 were significantly higher in the skin of the three patients, as compared to psoriasis-like reactions to anti-TNF-α, and similar to mRNA levels detected in chronic psoriasis plaques (**Figure 4A**). In line with previous studies (6, 8), aberrant IL-6 and TNF-α mRNA amounts were detected in the immune-related reactions to anti-PD-1 (**Figure 4A**). Finally, the analysis of selected innate immunity molecules showed that the type I IFN-β and the lymphotoxins LT-α and LT-β (belonging to the TNF cytokine family) mRNA are poorly expressed in psoriasis lesions induced by anti-PD-1, as compared to anti-TNF-α-induced psoriasis (**Figure 4B**).

### 3.3 ADAMTSL5 psoriasis autoantigen is overexpressed in psoriasis lesions induced by nivolumab treatment

**Figure 5:**
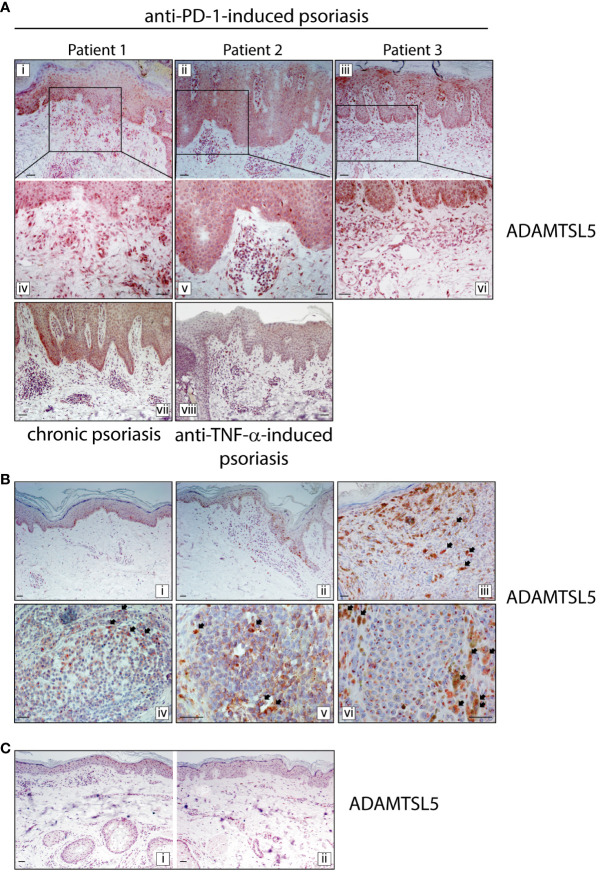
ADAMTSL5 psoriasis autoantigen is overexpressed in psoriasis lesions induced by nivolumab treatment, as well as in melanoma tissues. **(A)** Immunohistochemistry for ADAMTSL5 was conducted on psoriasis lesions induced by anti-PD-1 (Patient 1, 2 and 3), and compared to those present in chronic plaque-type psoriasis (*n*=6 patients) and in psoriasis induced by anti-TNF-α therapy (*n*=3). ADAMTSL5 immunoreactivity was mainly present in paradoxical psoriasis to anti-PD-1, in keratinocytes and in scattered basal cells with the morphology of melanocytes. It was also highly expressed in most dermal infiltrating cells, localized in the papillary and mid dermis, as well in perivascular and endothelial cells. Panels iv, v and vi represent the insets of panels i, ii, and iii, respectively, at a magnification of x200. ADAMTSL5 expression pattern observed in psoriasis to anti-PD-1 was similar to that observed in plaque-type psoriasis (vii). Anti-TNF-α -induced psoriasis lesions were negative (viii). **(B)** For patient 2, ADAMTSL5 was also detected in non-lesional (3-cm distant area from tumor lesion) (i), perilesional (ii) and lesional (iii-v) skin specimens obtained from the primary melanoma and melanoma skin metastasis (vi). Numerous ADAMTSL5^+^ melanophages/macrophages infiltrated melanoma tissues (arrows, iii-vi). **(C)** ADAMTSL5 expression was absent in both perilesional (i) and lesional (ii) skin of anti-PD-1-induced vitiligo developed by patient 2 after nivolumab treatment. For each specimen, one out of three representative staining is shown. Scale bars, 40 μm.

We next evaluated the expression of the melanocyte- and keratinocyte-derived autoantigen ADAMTSL5, whose expression has been found to be dysregulated in psoriasis (13, 14) and in many cancer types, including melanoma (28). Immunohistochemistry analysis carried out on immune-related psoriasis induced by anti-PD-1 treatment revealed a strong positivity for ADAMTSL5 antigen in skin lesions of all three patients (**Figure 5**). Immunoreactivity was mainly present in keratinocytes throughout the epidermis and in scattered basal cells with the morphology of melanocytes (**Figure 5A**). ADAMTSL5 was also highly expressed in most dermal infiltrating cells, expectedly DCs and macrophages, localized in the papillary and mid dermis, as well in perivascular and endothelial cells (**Figure 5A**). Non-lesional areas adjacent to developed psoriasiform reactions showed ADAMTSL5 immunoreactivity exclusively confined to melanocytes (not shown). ADAMTSL5 expression pattern observed in immune-related psoriasis induced by anti-PD-1 was similar to that observed in plaque-type psoriasis (**Figure 5A**), even though ADAMTSL5 antigen was not always detectable in all psoriasis specimens (3 of 6 psoriasis patients were ADAMTSL5^+^). None of the three paradoxical psoriasis to anti-TNF-α showed ADAMTSL5 positivity (**Figure 5A**).

Finally, for patient 2, we performed ADAMTSL5 immunohistochemistry on skin specimens obtained from lesional, perilesional and 3-cm distant areas of the primary melanoma, from melanoma skin metastasis and anti-PD-1-induced vitiligo. As shown in **Figure 5B**, ADAMTSL5 was expressed in most melanoma cells of primary tumor, with different intensity of staining. ADAMTSL5 immunoreactivity could be weak, moderate, or intense and localized in cytoplasmic foci/granules. An intense staining was also observed in perilesional area neighboring primary melanoma, specifically in melanocytes undergoing transformation and in few infiltrating dermal cells (**Figure 5B**, panel ii). Conversely, in the area 3-cm distant from the margin of tumor, ADAMTSL5 positivity was only found in melanocytes (**Figure 5B**, panel i). ADAMTSL5 staining was also observed in melanoma skin metastasis of patient 2, with an intense and granular positivity in the cytoplasm (**Figure 5B**, panel vi). Of note, we could detect numerous ADAMTSL5^+^ melanophages/macrophages infiltrating tumoral areas and characterized by a strong accumulation of melanosomes ingested from neighboring melanocytes (**Figure 5A**, panel iii-vi). These observations suggest that ADAMTSL5 protein is produced by tumor cells in significant amounts and undergoes subsequent uptake by melanophage/macrophage subpopulation. Finally, in vitiligo specimens of patient 2, ADAMTSL5 expression was absent in both perilesional and lesional skin, except for few cells infiltrating the dermis (**Figure 5C**).

### 3.4 SNP characterization in anti-PD-1-treated patients developing immune-mediated psoriasis reactions

In order to understand whether immune-related psoriasis development in patients undergone nivolumab treatment had a genetic basis, we analyzed by high-throughput NGS a panel of SNPs predisposing to psoriasis. Among them, we studied SNPs frequent in psoriatic population, such as polymorphisms in *HLA-C, HLA-B, ERAP1, PSORS1C3, MICA* and other genes of *PSORS1* locus. We also analyzed genetic variants of pathogenic cytokines, receptors, signal transducers and regulators of cytokine signaling (i.e., TNF-α, IL-17F, IL-17RA, IL-23R, IL-12B, TNFSF15, TNFRSF1B, TRAF3IP2, TNAIP3, NFKBIZ, SOCS1 and Tyk2), as well as SNPs in genes encoding skin-barrier proteins (i.e., *CDSN, CCHCR1*) and innate immunity molecules (i.e., IFIH1, DDX58 and LTA) (**Supplementary Table 1**). All patients showed variants of ERAP1 and HLA-C region, either in homozygosis or heterozygosis (**Supplementary Table 1**). SNP pattern in *ERAP1* were specific for each patient and affected both exons and intronic regions of the gene. Several variants in HLA-C promoter were found in patient 1, who showed concomitant SNPs in HLA-C upstream region (~35 kb) (rs12191877, rs17192519, rs198874, rs17198895, rs17192526, rs4406273, rs2524095, rs2853922) (**Supplementary Table 1**). Patient 2 showed only two SNPs in HLA-C upstream region (rs9264942, rs10484554), whereas patient 3 showed four critical SNPs in the 3’UTR of HLA-C (rs1130538, rs1130580, rs1130592, rs1094) and five SNPs in HLA-C upstream region (rs9348865, rs9264944, rs9264946, rs76703505, rs3094691). Interestingly, patient 2 showed three SNPs (rs2523473, rs2428476, rs28366116) in homozygosis in *MICA* (**Supplementary Table 1**). A specific SNP pattern was observed for each patient, even though all patients carried out numerous SNPs in genes involved in skin barrier function, namely in *LCE 3A-B*, *LCE1C*, *CDSN* and *CCHCR1* (**Supplementary Table 1**). Concerning variants of genes encoding cytokines, receptors, signal transducers and regulators of cytokine signaling, patient 1 showed SNPs in *TNFRSF1B* (five SNPs of which four were in homozygosis), *RUNX3* (rs7536201), *SLC12A8* (rs651630), *TNIP1* (three SNPs in heterozygosis), *IL12B* (rs3213094 and rs2546890), *SOCS1* (rs431918 in homozygosis), *FBXL19* (rs10782001 in homozygosis) and *IL17RA* (rs4819553 and rs4819958) (**Supplementary Table 1**). Patient 1 and 2 showed SNPs in *TNFA* (rs3093662 in patient 1 and rs3093661 in patient 2) and in *TNFRSF1A* (rs767455 in both patients). Patient 2 and 3 shared SNPs in *NFKBIZ* encoding the IKB-ζ signal transducer of IL-17 (rs595788, rs9881690, rs7625614 in patient 2 and rs9818678 in patient 3), *TNFAIP3* (rs582757 in both patients and rs610604 in patient 3). Patient 1 and 3 showed variants of *IL-23R* (rs12567033 in patient 1 and rs2201841 in patient 3), *TRAF3IP2* (rs33980500 and rs76228616 in patient 1 and rs71562288 and rs240993 in patient 3) and of *TNFRSF15* (rs6478109 in both patients). Patient 3 only carried out two SNPs in *CTLA4* (rs231721 and rs3087243, both in homozygosis) and in *RUNX1* (rs2834760) (**Supplementary Table 1**). All patients shared SNPs in *TNFSF15*, a gene encoding a cytokine belonging to the TNF ligand family.

## 4 Discussion

Psoriasis pathogenesis involves both innate and adaptive immunity responses, which are overactive in different clinical phases of the disease and characterized by specific patterns of inflammation. Innate immunity processes predominate in the early phase of psoriasis development, with pDC, neutrophils and mast cells being abundant in skin lesions. Conversely, adaptive immune responses driven by mDCs and T lymphocytes, mostly IL-17- and IFN-γ-producing CD8^+^ T cells, are typical of chronic, stable psoriasis (10, 29–31).

In this study, we found that immune-related psoriasis evoked by anti-PD-1 therapy in three patients affected by malignancies strongly resembles chronic psoriasis. In fact, by comparing skin lesions of immune-related psoriasis to anti-PD-1 with chronic plaque-type psoriasis and psoriasiform reactions to anti-TNF-α, we observed a prominent infiltrate of CD8^+^ T cells and CD11c^+^ DC, at levels and patterns of distribution of stable psoriasis, together with CD15^+^ neutrophils accumulating in sub corneal aggregates. In parallel, the mRNA expression levels of psoriasis-related cytokines, such as IL-17A, IL-23, IFN-γ and IL-22 greatly increased in immune-related skin reactions to anti-PD-1. Conversely, the immunological patterns typical of paradoxical psoriasis by anti-TNF-α and acute psoriasis, including overexpression of innate immunity molecules and dermal infiltration of BDCA^+^ pDC, CD15^+^ neutrophils, and c-kit/CD117^+^ mast cells could not be found.

Concerning mechanisms involved in anti-PD-1-induced psoriasis reactions, Tanaka R et al. described the contribution of CD8^+^ T cells, whose pathogenicity has been related to enhanced IL-6, IL-23 and IL-17A production. These findings were obtained by blocking IL-6 receptor in IMQ-induced psoriasis reactions in mice genetically modified and lacking PD-1 expression specifically in CD8^+^ T cells (8). We confirmed the observation that CD8^+^ T cells strongly infiltrate psoriasis-like lesions of patients undergone anti-PD-1 therapy, with a preferential accumulation of these cells in the epidermal compartment. CD8^+^ T cells were in close contact with keratinocytes and distributed as in chronic psoriasis, where an aberrant crosstalk *via* MHC class I molecules and (auto)antigen presentation by keratinocytes occurs (10). We also found that IL-6 and TNF-α are aberrantly expressed in psoriasis reactions induced by anti-PD-1, at levels comparable with chronic psoriasis. Increased IL-6 production in patients affected by psoriasis has been extensively described (32) and correlated to several pathological effects within affected tissues, including differentiation of type-17 lymphocytes and dampening of regulatory T (T_reg_) cell function (33). In chronic psoriasis, IL-6, abundantly released by Th17 cells, sustains deleterious loops leading to Th17/T_reg_ unbalance (34, 35). Of note, IL-6-induced effects, which are deleterious in patients affected by psoriasis, could be instead protective in the cancer context and limit tumor growth and expansion, by promoting expansion of cytotoxic IL-17-producing T cells and preventing immune suppression by T_reg_. However, although melanoma microenvironment can provide an optimal cytokine milieu for Th17 recruitment/expansion by expressing high IL-6 (36), and IFN-γ-releasing Th17 cells show antitumor effects through recruitment of cytotoxic CD8^+^ T cells (37), IL-17A-expressing cells were generally few around the primary melanoma lesions (38). It would be of interest to investigate whether Th17 lymphocytes are present in melanoma lesions in patients developing cutaneous ircAEs.

Here, we also show that ADAMTSL5 antigen was strongly expressed in psoriasis lesions of the three oncological patients, at similar expression levels and localization of chronic plaque-type psoriasis. However, ADAMTSL5 was not detectable in all patients affected by plaque psoriasis, confirming that different (auto)antigens can elicit adaptive immune responses in the disease (39, 40). In addition, ADAMTSL5 was not expressed in psoriasis reactions to anti-TNF-α, in line with previous findings showing an overactivation of innate immunity, and not adaptive responses, in these conditions (17, 41). Importantly, in patient 2, ADAMTSL5 expression was not only found in psoriasis skin lesions but also in most melanoma cells of the primary tumor and in perilesional areas, specifically in melanocytes undergoing transformation. ADAMTSL5 also accumulated in melanophages/macrophages infiltrating tumoral and peritumoral areas, indicating that ADAMTSL5 protein is produced by melanoma cells in significant amounts and undergoes subsequent uptake by melanophages. These latter cells are macrophage-tumor hybrids, which have been related to the tumorigenicity and metastatic potential of melanoma (42). In fact, melanophages have a strong migratory capacity *in vitro* and can spread to skin-draining lymph nodes (43), where cross-presentation of melanoma antigens by DCs or other antigen presenting cells to naïve T cells typically occurs (44). The presence of ADAMTSL5 antigen in primary melanoma, as well as in cutaneous and lymph node metastasis (data not shown) suggest that ADAMTSL5-specific T-cell responses can be driven in melanoma patients, with the possibility of induction of immune responses triggering/exacerbating psoriasis in permissive conditions (anti-PD-1 treatment). Besides melanoma, ADAMTSL5-specific immune responses could also be induced in other cancer types, being ADAMTSL5 dysregulated in a wide variety of malignant tumors, including lung cancer and hepatocarcinoma (28, 45). In hepatocarcinoma condition, ADAMTSL5 overexpression derives from hypermethylation of *ADAMTSL5* gene body and localizes in hepatocarcinoma cells and in macrophages of necrotic areas of tumors. Importantly, ADAMTSL5 confers tumorigenicity by upregulating oncogenic inputs (i.e., MET, EGFR, PDGFRβ, IGF1Rβ, FGFR4), and its abrogation increases sensitivity of tumor cells to clinically relevant drugs (45).

In psoriasis patients, specific immune responses induced by ADAMTSL5 autoantigen are represented primarily by IL-17A-producing CD8^+^ T cells and directed against melanocytes and surrounding keratinocytes (12). ADAMTSL5 recognition occurs through HLA-Cw6 class I molecule, which presents peptide ligands by recognizing Vα3S1/Vβ13S1 TCR on CD8^+^ T cells (12). It would be interesting to examine whether ADAMTSL5 might represent a potential antigen also for melanoma and other malignancies, recognized by CD8^+^ T cells with specific TCR repertoire. There is the possibility that anti-PD-1-induced immune responses specific for ADAMTSL5 could be responsible for both anti-PD-1-induced psoriasis, and for an effective immune response against melanoma cells. The favorable prognosis of patients 1 and 2 after nivolumab treatment is in line with this hypothesis since both of them showed high expression of the ADAMTSL5 autoantigens. Instead, the poor prognosis of patient 3 could depend on the short-term treatment with nivolumab and concomitant immunosuppressive therapies for limiting psoriasis and immune-related responses.

Psoriasis disease manifestation occurs only in a portion of subjects undergone anti-PD-1 therapies for advanced solid tumors, with an incidence rate of ~ 4.3% in patients developing ircAEs (46). Therefore, it is reasonable to speculate the influence of genetic factors predisposing to immune-related psoriasis to anti-PD-1, and specifically being involved in driving and amplifying type-17 and type-1 adaptive immune responses. Indeed, a genetic predisposition to other types of psoriasis reactions induced by therapies with immunomodulators have been described (17, 47). To date, no evidence correlating the presence of SNPs and the development of psoriasis in patients in treatment with anti-PD-1 exist. In our study, we found that all patients carried numerous allelic variants in *HLA-C*. None of the patients showed the *HLA-Cw6* susceptibility allele, even though other SNPs possibly involved in the regulation of HLA-C expression levels (i.e., *HLA-C* promoter, *HLA-C* 3’UTR and *HLA-C* upstream region) were found. These findings are important since specific HLA-C haplotypes have been correlated with the clinical course of psoriasis disease, and over one-hundred SNPs of *HLA-C* genic and intergenic region have been described in patients (48). In addition, evidence has emerged for the presence of susceptibility alleles of other MHC class I genes and regulatory regions, potentially influencing HLA expression in the psoriatic population. They include polymorphic regions in proximity to *MICA*, which encodes MHC class I-related proteins with potential immunological functions on IL-17A-producing and CD8^+^ T cells (49). These latter polymorphisms were only found in patient 2, who showed the most favorable response to anti-PD-1 treatment. Concomitantly, oncological patients carried allelic variants in the *ERAP1* gene, consistently with the presence CD8^+^ T-cell responses in psoriasis reactions to anti-PD-1. Interestingly, different ERAP1 haplotypes controlling the likelihood and strength of the immune response have been identified (50). Among them, the haplotype 2 (rs26653) can control the autoimmune response against melanocytes in psoriasis by generating ADAMTSL5 antigen epitopes (50). All patients show specific ERAP1 haplotypes, which may determine the generation and different amounts of certain autoantigens for HLA-class I presentation with the subsequent risk of autoimmune CD8^+^ T-cell activation (50). Other than having a role in MHC class I antigen presentation, ERAP1 is involved in the activation of inflammasome pathways and production of cytokines and chemokines involved in psoriasis development (i.e., IL-6, TNF-α, and CCL2) (51).

Although all patients carried numerous SNPs in genes involved in skin barrier function, a specific SNP pattern in *LCE 3A-B* and *LCE1C*, as well as in *CDSN* and *CCHCR1*, was observed for each patient. In particular, several SNPs were identified in *LCE* gene cluster, located in the epidermal differentiation complex of *PSORS4* locus and encoding structural proteins with a role in epithelial barrier formation, as well as peptides with antimicrobial activity (52). All LCE allelic variants are likely involved in the pathogenic responses induced by IL-17 in psoriatic keratinocytes, in terms of terminal differentiation and proliferation, two processes contributing to epidermal acanthosis typical of psoriatic lesions (10). Previous studies identified several conserved, noncoding elements within *LCE* intergenic region exhibiting dynamic regulatory activity and coordinating LCE expression in differentiating or proliferating cells (53). In all patients, we found SNPs located between *LCE3B* and *LCE3A*, in an intergenic region potentially involved in the regulation of expression levels of LCE3 genes. This genomic sequence could have regulatory functions like the entire *LCE3B/C* region, whose deletion leads to increased LCE3A mRNA expression in psoriatic skin under the influence of Th1 and Th17 cytokines (54).

We found few SNPs in exon 2 of *CDSN* overlapping with *PSORS1C1*, which can give rise to missense variants strongly impacting on corneocyte adhesion and skin desquamation, as well as associating with increased risk of psoriasis severity (55). The potential effects of SNPs on PSORS1C1 expression and function in patients is unpredictable.

Concerning *CCHCR1*, we found several SNPs in the three patients, with different distribution in introns and exons of the gene. In patient 1, we detected SNPs potentially leading to amino acid substitution in exon 4 (rs130065, rs130066, rs130076), exon 14 (rs130079) and in exon 18 (rs1576) and two SNPs in intron 10 (rs746647, rs2240065). Patient 2 and 3 showed two SNPs in homozygosis in *CCHCR1*, both present in intron 13 (rs3094226, rs2073719). Although these latter two SNPs are irrelevant for amino acid composition of CCHCR1 protein, their potential regulatory function of CCHCR1 mRNA expression cannot be excluded. The consequence of the SNP presence in CCHCR1 in patients could be multiple, depending on SNP presence and haplotypes, which give raise different CCHCR1 mRNA and protein variants (56). CCHCR1 influences keratinocyte proliferation by regulating cytoskeleton as well as other processes including RNA surveillance and transport (57). The function of CCHCR1 isoforms in psoriasis together with the IL-17-dependent mechanisms regulating their expression pattern in psoriatic skin remains to be elucidated. NGS analysis of variants of genes encoding cytokines, receptors, signal transducers and regulators of cytokine signaling showed that all patients carried SNPs in *TNFSF15*, a gene encoding TL1A. TL1A is a TNF-like protein overexpressed in psoriasis, that competitively binds to death receptor 3, providing stimulatory signal for proliferation, activation, apoptosis and cytokine in effector immune cells (58). Interestingly, patient 1 had a peculiar genetic pattern, and differently from the other two patients carried several SNPs predisposing to psoriasis. These SNPs were present in *TNFRSF1B, RUNX3, SLC12A8, TNIP1, IL12B, SOCS1, FBXL19* and *IL17RA*, and were associated with the severity and early onset of the disease (59). Indeed, patient 1 developed the most severe form of psoriasis among the three patients and had a personal history for the disease. On the other hand, patient 2 and 3 shared SNPs in *NFKBIZ* and in *TNFAIP3*, encoding the IL-17/TRAF6 signaling regulators IKB-ζ and A20, respectively, whose genetic variability could contribute to immune dysregulation and chronic inflammation in psoriasis lesions (60). Patient 3 only showed polymorphisms in a region downstream of *CTLA4*, which have been previously associated to paradoxical psoriasis to anti-TNF-α (61). In the future, it will be necessary to extend the analysis of psoriasis-related SNPs to a largest cohort of oncological patients developing psoriasiform reactions to anti-PD-1, but also in a population successfully responding to the treatment, to identify differences in the genetic background of the patients. The identification of genetic biomarkers correlating with an adverse response to anti-PD-1 therapy will be useful to predict the risk of developing immune-related psoriasis.

In conclusion, our study shows that immune-related psoriasis induced by anti-PD-1 therapy in three oncological patients has immunological features common to chronic phase psoriasis, mainly characterized by cellular and molecular players of adaptive immunity. Among them, CD8^+^ T cells were found in the epidermis of skin lesions and in close contact with keratinocytes, consistently with the local overexpression and exposure of ADAMTSL5 psoriasis autoantigen. The latter has also been found in melanoma tissues, suggesting a possible role of ADAMTSL5 in evoking T-cell responses in common with psoriasis. The genetic susceptibility of patients to develop immune responses typical of psoriasis would confirm this possibility. It will be important to evaluate the presence and features of ADAMTSL5-specific T-cell responses in oncological patients developing chronic psoriasis following anti-PD-1 therapies, and to assess whether these responses may concomitantly trigger the onset of psoriasis and the protection against tumor.

## Data availability statement

The datasets presented in this study can be found in online repositories. The names of the repository/repositories and accession number(s) can be found below: https://www.ncbi.nlm.nih.gov/sra/PRJNA107495558, PRJNA1074955.

## Ethics statement

The studies involving humans were approved by the Ethics committee of Istituto Dermopatico dell'Immacolata Hospital, Rome, Italy (Prot. CE 475/2016). The studies were conducted in accordance with the local legislation and institutional requirements. The participants provided their written informed consent to participate in this study. Written informed consent was obtained from the individual(s) for the publication of any potentially identifiable images or data included in this article.

## Author contributions

MM: Conceptualization, Data curation, Formal analysis, Investigation, Methodology, Software, Validation, Writing – review & editing. MC: Conceptualization, Formal analysis, Investigation, Methodology, Validation, Writing – review & editing. GS: Formal analysis, Software, Writing – review & editing. CS: Formal analysis, Methodology, Validation, Writing – review & editing. VF: Methodology, Writing – review & editing. SP: Resources, Writing – review & editing. FG: Resources, Writing – review & editing. SR: Formal analysis, Writing – review & editing. SM: Conceptualization, Supervision, Writing – review & editing. CF: Conceptualization, Supervision, Writing – review & editing. CA: Conceptualization, Data curation, Formal analysis, Funding acquisition, Resources, Supervision, Writing – original draft, Writing – review & editing.
