# Supplementary material for: Identification of immunological patterns characterizing immune-related psoriasis reactions in oncological patients in therapy with anti-PD-1 checkpoint inhibitors
Source: Front Immunol. 2024 Mar 1;15:1346687. doi: 10.3389/fimmu.2024.1346687 (PMC10940473; doi:10.3389/fimmu.2024.1346687)
Supplement: Supplementary file 1 [file Table_1.docx]

**Supplementary Table 1. SNP status in patients**

| **Antigen presentation** | | | |  |  |  |  |
| --- | --- | --- | --- | --- | --- | --- | --- |
| **Genome position** | **Gene** | **SNP name** | **dbSNP ID** | **Patient 1** | **Patient 2** | **Patient 3** | **MAF** |
| Chr5:96101944 | ERAP1 | ERAP1_v1 | rs27524 | 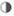 | 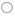 | 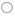 | 0.6296 |
| Chr5:96101959 | ERAP1 | ERAP1_v2 | rs27525 | 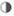 | 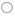 | 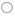 | 0.4532 |
| Chr5:96118852 | ERAP1 | ERAP1_v3 | rs27044 | 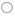 | 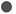 | 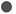 | 0.7158 |
| Chr5:96118866 | ERAP1 | ERAP1 | rs17482078 | 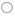 | 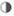 | 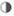 | 0.1837 |
| Chr5:96119044 | ERAP1 | ERAP1_v12 | rs62364748 | 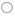 | 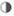 | 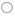 | 0.0781 |
| Chr5:96119055 | ERAP1 | ERAP1_v13 | rs11738810 | 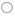 | 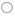 | 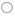 | 0.1061 |
| Chr5:96124330 | ERAP1 | ERAP1_v4 | rs30187 | 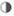 | 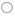 | 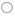 | 0.6491 |
| Chr5:96124447 | ERAP1 | ERAP1_v5 | rs30186 | 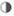 | 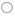 | 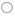 | 0.7886 |
| Chr5:96124453 | ERAP1 | ERAP1_v6 | rs11743410 | 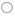 | 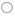 | 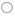 | 0.1245 |
| Chr5:96139066 | ERAP1 | ERAP1_v20 | rs10062964 | 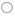 | 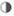 | 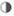 | 0.1724 |
| Chr5:96139250 | ERAP1 | ERAP1_v21 | rs26653 | 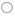 | 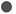 | 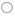 | 0.2250 |
| Chr6:31155539 | PSORS1C3 | PSORS1C3_v3 | rs4713443 | 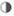 | 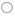 | 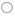 | 0.3849 |
| Chr6:31155548 | PSORS1C3 | PSORS1C3_v4 | rs4713444 | 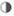 | 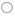 | 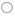 | 0.4315 |
| Chr6:31155659 | PSORS1C3 | PSORS1C3_v6 | rs9263845 | 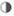 | 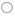 | 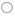 | 0.1594 |
| Chr6:31155670 | PSORS1C3 | PSORS1C3_v7 | rs9263846 | 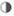 | 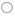 | 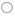 | 0.1781 |
| Chr6:31155785 | PSORS1C3 | PSORS1C3_v1 | rs1265181 | 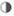 | 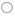 | 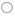 | 0.2115 |
| Chr6:31236679 | HLA-C | HLA-C_v5 | rs1130580 | 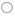 | 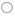 | 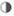 | 0.3358 |
| Chr6:31236690 | HLA-C | HLA-C_v6 | rs1130592 | 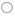 | 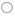 | 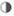 | 0.3189 |
| Chr6:31236722 | HLA-C | HLA-C_v7 | rs1094 | 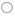 | 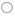 | 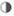 | 0.3231 |
| Chr6:31236853 | HLA-C | HLA-C_v14 | rs1065711 | 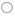 | 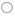 | 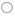 | 0.1314 |
| Chr6:31239108 | HLA-C | HLA-Cw6 | rs1131118 | 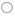 | 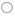 | 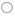 | 0.3143 |
| Chr6:31239506 | HLA-C | HLA-C exon2 | rs1050414 | 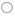 | 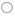 | 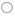 | 0.1201 |
| Chr6:31241077 | HLA-C region | HLA-C_promoter_v4 | rs35976302 | 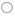 | 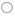 | 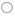 | 0.1272 |
| Chr6:31241092 | HLA-C region | HLA-C_promoter_v5 | rs2523599 | 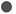 | 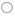 | 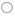 | 0.3645 |
| Chr6:31241109 | HLA-C region | HLA-C_promoter1 | rs13191343 | 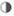 | 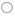 | 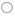 | 0.1394 |
| Chr6:31241127 | HLA-C region | HLA-C_promoter2 | rs13207315 | 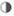 | 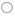 | 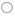 | 0.1429 |
| Chr6:31241182 | HLA-C region | HLA-C_ promoter3 | rs6900444 | 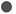 | 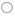 | 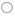 | 0.4937 |
| Chr6:31241207 | HLA-C region | HLA-C_promoter_v7 | rs6900458 | 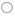 | 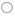 | 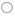 | 0.2140 |
| Chr6:31241215 | HLA-C region | HLA-C_promoter_v8 | rs58019823 | 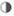 | 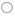 | 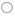 | 0.1239 |
| Chr6:31241270 | HLA-C region | HLA-C_promoter_v10 | rs6900323 | 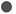 | 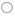 | 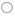 | 0.2205 |
| Chr6:31241294 | HLA-C region | HLA-C_promoter_v11 | rs2524087 | 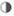 | 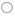 | 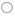 | 0.0413 |
| Chr6:31241311 | HLA-C region | HLA-C_promoter_v12 | rs2524086 | 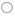 | 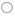 | 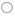 | 0.1476 |
| Chr6:31241370 | HLA-C region | HLA-C_promoter_v14 | rs6923313 | 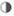 | 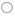 | 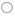 | 0.3589 |
| Chr6:31251924 | HLA-C region | HLA-Cw6_LD1 | rs12189871 | 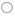 |  |  | 0.0904 |
| Chr6:31252747 | HLA-C region | HLA-C_v1  intergenic region | rs9348862 |  |  |  | 0.0859 |
| Chr6:31252882 | HLA-C region | HLA-C_v2  intergenic region | rs9368670 |  |  |  | 0.0841 |
| Chr6:31252925 | HLA-C region | HLA-Cw6_LD2 | rs12191877 |  |  |  | 0.13253 |
| Chr6:31265940 | HLA-C region | HLA-C | rs17192519 |  |  |  | 0.3085 |
| Chr6:31265989 | HLA-C region | HLA-C | rs17198874 |  |  |  | 0.3079 |
| Chr6:31266015 | HLA-C region | HLA-C | rs17198888 |  |  |  | 0.1773 |
| Chr6:31266033 | HLA-C region | HLA-C | rs17198895 |  |  |  | 0.3056 |
| Chr6:31266041 | HLA-C region | HLA-C | rs17192526 |  |  |  | 0.4381 |
| Chr6:31266085 | HLA-C region | HLA-C_v4 | rs17192533 |  |  |  | 0.1028 |
| Chr6:31266090 | HLA-C region | HLA-Cw6_LD3 | rs4406273 |  |  |  | 0.0775 |
| Chr6:31266117 | HLA-C region | HLA-C_v5 | rs2524095 |  |  |  | 0.5689 |
| Chr6:31266151 | HLA-C region | HLA-C_v6 | rs7761855 |  |  |  | 0.0633 |
| Chr6:31266190 | HLA-C region | HLA-C_v7 | rs2853922 |  |  |  | 0.6029 |
| Chr6:31274380 | HLA-C region | HLA-Cw6_LD4 | rs9264942 |  |  |  | 0.3654 |
| Chr6:31274449 | HLA-C region | HLA-C_v9 | rs35647108 |  |  |  | 0.0671 |
| Chr6:31274513 | HLA-C region | HLA-C_v10 | rs6931873 |  |  |  | 0.2109 |
| Chr6:31274555 | HLA-C region | HLA-Cw6_LD5 | rs10484554 |  |  |  | 0.1442 |
| Chr6:31274582 | HLA-C region | HLA-C_v13 | rs9348865 |  |  |  | 0.3769 |
| Chr6:31274586 | HLA-C region | HLA-C_v15 | rs9348865 |  |  |  | 0.3769 |
| Chr6:31274619 | HLA-C region | HLA-C_v16 | rs9264944 |  |  |  | 0.2350 |
| Chr6:31274634 | HLA-C region | HLA-C_v17 | rs9264946 |  |  |  | 0.2053 |
| Chr6:31274666 | HLA-C region | HLA-C | rs76703505 |  |  |  | 0.0875 |
| Chr6:31274693 | HLA-C region | HLA-C | rs3094691 |  |  |  | 0.4678 |
| Chr6:31324996 | HLA-B | HLA-B_v12 | rs2596490 |  |  |  | 0.4462 |
| Chr6:31325056 | HLA-B | HLA-B_v22 | rs2596487 |  |  |  | 0.1829 |
| Chr6:31344471 | FGFR3P1 region | FGFR3P1 region_v4 | rs3997983 |  |  |  | 0.3476 |
| Chr6:31344490 | FGFR3P1 region | FGFR3P1 region_v6 | rs3957111 |  |  |  | 0.1235 |
| Chr6:31344511 | FGFR3P1 region | FGFR3P1 region_v1 | rs28366075 |  |  |  | 0.0897 |
| Chr6:31344571 | FGFR3P1 region | FGFR3P1 region_v2 | rs28366076 |  |  |  | 0.0877 |
| Chr6:31344583 | FGFR3P1 region | FGFR3P1 region_v3 | rs13202464 |  |  |  | 0.0696 |
| Chr6:31344626 | FGFR3P1 region | FGFR3P1 region_v8 | rs9266596 |  |  |  | 0.2264 |
| Chr6:31344657 | FGFR3P1 region | FGFR3P1 region_v9 | rs2844546 |  |  |  | 0.2931 |
| Chr6:31344670 | FGFR3P1 region | FGFR3P1 region_v10 | rs2523637 |  |  |  | 0.3456 |
| Chr6:31361897 | MICA region | MICA region_v2 | rs2523473 |  |  |  | 0.3505 |
| Chr6:31361987 | MICA region | MICA region_v4 | rs35026345 |  |  |  | 0.3019 |
| Chr6:31362010 | MICA region | MICA region_v5 | rs34464243 |  |  |  | 0.2723 |
| Chr6:31362069 | MICA region | MICA region_v6 | rs67284927 |  |  |  | 0.1341 |
| Chr6:31362120 | MICA region | MICAregion_v1 | rs66609536 |  |  |  | 0.2539 |
| Chr6:31362159 | MICA | MICA_v4 | rs2428476 |  |  |  | 0.0879 |
| Chr6:31362166 | MICA | MICA_v5 | rs28366116 |  |  |  | 0.0879 |
| Chr6:31376928 | MICA | MICA_v1 | rs2523497 |  |  |  | 0.4312 |
| Chr6:31377047 | MICA | MICA_v2 | rs6910087 |  |  |  | 0.1305 |
| Chr6:31377100 | MICA | MICA_11 | rs3032981 |  |  |  | 0.1684 |
| Chr6:31431691 | HCP5 | HCP5_v6 | rs2255221 |  |  |  | 0.0818 |
| Chr6:31431723 | HCP5 | HCP5_v7 | rs2255223 |  |  |  | 0.0415 |
| Chr6:31431757 | HCP5 | HCP5_v8 | rs11752262 |  |  |  | 0.0714 |
| Chr6:31431813 | HCP5 | HCP5_v9 | rs3130907 |  |  |  | 0.1051 |
| Chr6:31431820 | HCP5 | HCP5_v2 | rs2243621 |  |  |  | 0.1611 |
| Chr6:31431874 | HCP5 | HCP5_v3 | rs2395030 |  |  |  | 0.0474 |
| Chr6:31431911 | HCP5 | HCP5_v10 | rs78490730 |  |  |  | 0.0017 |
| Chr6:31461372 | MICB-DT | MICB-DT_v1 | rs2507971 |  |  |  | 0.6052 |
| Chr6:31461492 | MICB-DT | MICB-DT_v2 | rs9267325 |  |  |  | 0.1409 |
| Chr6:31461509 | MICB-DT | MICB-DT_v4 | rs2516413 |  |  |  | 0.2891 |
| Chr6:31461558 | MICB-DT | MICB-DT_v5 | rs3094006 |  |  |  | 0.2653 |
| Chr6:31461608 | MICB-DT | MICB-DT_v6 | rs2534687 |  |  |  | 0.3360 |

| **Skin barrier function** | | | | |  | |  |  |  |
| --- | --- | --- | --- | --- | --- | --- | --- | --- | --- |
| **Genome position** | **Gene** | **SNP name** | **dbSNP ID** | | **Patient 1** | | **Patient 2** | **Patient 3** | **MAF** |
| Chr1:152550018 | LCE region | LCE_v1 | rs4085613 | |  | |  |  | 0.6370 |
| Chr1:152551276 | LCE3B region | LCE3B | rs4112788 | |  | |  |  | 0.6369 |
| chr1:152551325 | LCE3B region | LCE3B_v2 | rs4112787 | |  | |  |  | 0.3505 |
| chr1:152551547 | LCE3D region | LCE3D | rs61813877 | |  | |  |  | 0.3435 |
| Chr1:152590187 | LCE region | LCE_v2 | rs6677595 | |  | |  |  | 0.6361 |
| Chr1:152590444 | LCE3A-B intergenic region | LCE3A-B_v4 | rs6701307 | |  | |  |  | 0.3916 |
| Chr1:152590886 | LCE3A-B intergenic region | LCE3A-B_v5 | rs4845447 | |  | |  |  | 0.3921 |
| Chr1:152590890 | LCE3A-B intergenic region | LCE3A-B_v2 | rs6701730 | |  | |  |  | 0.2454 |
| Chr1:152590943 | LCE3A-B intergenic region | LCE3A-B_v7 | rs4845448 | |  | |  |  | 0.3244 |
| Chr1:152591024 | LCE3A-B intergenic region | LCE3A-B_v9 | rs4845449 | |  | |  |  | 0.3925 |
| Chr1:152591142 | LCE region | LCE_v3 | rs1886734 | |  | |  |  | 0.6369 |
| Chr1:152591200 | LCE3A-B intergenic region | LCE3A-B_v10 | rs4845450 | |  | |  |  | 0.3881 |
| Chr1:152591953 | LCE3A-B intergenic region | LCE3A-B_v11 | rs4845453 | |  | |  |  | 0.4916 |
| Chr1:152591964 | LCE3A-B intergenic region | LCE3A-B_v12 | rs112857972 | |  | |  |  | 0.0136 |
| Chr1:152592184 | LCE region | LCE_v4 | rs4845454 | |  | |  |  | 0.6376 |
| Chr1:152593292 | LCE3A-B intergenic region | LCE3A-B_v1 | rs12030223 | |  | |  |  | 0.2539 |
| Chr1:152593307 | LCE3A-B intergenic region | LCE3A-B_v13 | rs6662989 | |  | |  |  | 0.3599 |
| Chr1:152593437 | LCE3A-B intergenic region | LCE3A-B_v14 | rs11205044 | |  | |  |  | 0.3931 |
| Chr1:152593549 | LCE region | LCE_v5 | rs10888503 | |  | |  |  | 0.6442 |
| Chr1:152778443 | LCE1C | LCE1C_v2 | rs12025125 | |  | |  |  | 0.1371 |
| Chr1:152778526 | LCE1C | LCE1C | rs6701216 | |  | |  |  | 0.1467 |
| Chr1:152778558 | LCE1C | LCE1C_v3 | rs6701221 | |  | |  |  | 0.1515 |
| Chr1:152778576 | LCE1C | LCE1C_v4 | rs4845488 | |  | |  |  | 0.4681 |
| Chr6:31084048 | CDSN | CDSN_v9 | rs3094216 | |  | |  |  | 0.2195 |
| Chr6:31084075 | CDSN | CDSN_v10 | rs3130982 | |  | |  |  | 0.4899 |
| Chr6:31084163 | CDSN | CDSN_v1 | rs3132554 | |  | |  |  | 0.5235 |
| Chr6:31084170 | CDSN | CDSN_v2 | rs1042127 | |  | |  |  | 0.1935 |
| Chr6:31084288 | CDSN | CDSN_v4 | rs1042126 | |  | |  |  | 0.5185 |
| Chr6:31084639 | CDSN | CDSN_v5 | rs4713436 | |  | |  |  | 0.1821 |
| Chr6:31084684 | CDSN | CDSN_v14 | rs3094215 | |  | |  |  | 0.4700 |
| Chr6:31084787 | CDSN | CDSN_v6 | rs707913 | |  | |  |  | 0.2139 |
| Chr6:31084792 | CDSN | CDSN_v7 | rs3130983 | |  | |  |  | 0.5186 |
| Chr6:31110391 | CCHCR1 | CCHCR1_v1 | rs1576 | |  | |  |  | 0.3461 |
| Chr6:31112737 | CCHCR1 | CCHCR1_v2 | rs130079 | |  | |  |  | 0.2491 |
| Chr6:31112899 | CCHCR1 | CCHCR1_v11 | rs3094226 | |  | |  |  | 0.2305 |
| Chr6:31112925 | CCHCR1 | CCHCR1_v3 | rs2073719 | |  | |  |  | 0.2393 |
| Chr6:31114182 | CCHCR1 | CCHCR1_v4 | rs746647 | |  | |  |  | 0.3220 |
| Chr6:31114335 | CCHCR1 | CCHCR1_v12 | rs2240066 | |  | |  |  | 0.0434 |
| Chr6:31114449 | CCHCR1 | CCHCR1_v14 | rs2240065 | |  | |  |  | 0.1921 |
| Chr6:31122315 | CCHCR1 | CCHCR1_v15 | rs130066 | |  | |  |  | 0.4133 |
| Chr6:31122330 | CCHCR1 | CCHCR1_v16 | rs130077 | |  | |  |  | 0.1519 |
| Chr6:31122482 | CCHCR1 | CCHCR1_v6 | rs130076 | |  | |  |  | 0.2185 |
| Chr6:31122500 | CCHCR1 | CCHCR1_v7 | rs130065 | |  | |  |  | 0.2083 |
| Chr6:31122502 | CCHCR1 | CCHCR1_v19 | rs130075 | |  | |  |  | 0.0527 |
| Chr9:110816795 | KLF4 region | KLF4_v2 | rs1369190 | |  | |  |  | 0.3718 |
| Chr9:110817020 | KLF4 region | KLF4 | rs10979182 | |  | |  |  | 0.4154 |
| **Innate immunity** | | | |  | |  | |  |  |
| **Genome position** | **Gene** | **SNP name** | **dbSNP ID** | **Patient 1** | | **Patient 2** | | **Patient 3** | **MAF** |
| Chr2:113739532 | IL36G | IL36G_v7 | rs6743744 |  | |  | |  | 0.0639 |
| Chr2:163124051 | IFIH1 | IFIH1 | rs1990760 |  | |  | |  | 0.6002 |
| Chr6:31540071 | LTA | LTA_v1 | rs1800683 |  | |  | |  | 0.3257 |
| Chr6:31540141 | LTA | LTA_v4 | rs2239704 |  | |  | |  | 0.4097 |
| Chr6:31540313 | LTA | LTA_v2 | rs909253 |  | |  | |  | 0.3326 |
| Chr6:31542308 | LTA region | LTA_v5 | rs1799964 |  | |  | |  | 0.2115 |
| Chr6:31542476 | LTA region | LTA_v6 | rs1800630 |  | |  | |  | 0.1526 |
| Chr6:31542482 | LTA region | LTA_v3 | rs1799724 |  | |  | |  | 0.1149 |
| Chr8:7273050 | DEFB4B | DEFB4B_v2 | rs73661358 |  | |  | |  | 0.1517 |
| Chr9:32534714 | DDX58 region | DDX58_v1 | rs34085293 |  | |  | |  | 0.1410 |
| Chr9:32534851 | DDX58 region | DDX58_v2 | rs657454 |  | |  | |  | 0.6309 |
| Chr19:10469975 | TYK2 | TYK2_v1 | rs12720356 |  | |  | |  | 0.0883 |
| Chr19:10472933 | TYK2 | TYK2_v2 | rs280519 |  | |  | |  | 0.5167 |
| Chr19:10475649 | TYK2 | TYK2_v3 | rs2304255 |  | |  | |  | 0.0758 |
| Chr19:10475652 | TYK2 | TYK2_v4 | rs2304256 |  | |  | |  | 0.2850 |
| Chr19:10475760 | TYK2 | TYK2_v9 | rs12720270 |  | |  | |  | 0.1634 |
| Chr20:30045393 | DEFB123 region | DEFB123 | rs6088273 |  | |  | |  | 0.6282 |

| **Cytokine-dependent pathways and T-cell signaling** | | | |  |  |  |  |
| --- | --- | --- | --- | --- | --- | --- | --- |
| **Genome position** | **Gene** | **SNP name** | **dbSNP ID** | **Patient 1** | **Patient 2** | **Patient 3** | **MAF** |
| Chr1:12252892 | TNFRSF1B | TNFRSF1B_v4 | rs1768642 |  |  |  | 0.2244 |
| Chr1:12252955 | TNFRSF1B | TNFRSF1B_v1 | rs1061622 |  |  |  | 0.2367 |
| Chr1:12253062 | TNFRSF1B | TNFRSF1B_v5 | rs5746026 |  |  |  | 0.0314 |
| Chr1:12267265 | TNFRSF1B | TNFRSF1B_v2 | rs1061624 |  |  |  | 0.5554 |
| Chr1:12267270 | TNFRSF1B | TNFRSF1B_v8 | rs5030792 |  |  |  | 0.0410 |
| Chr1:12267292 | TNFRSF1B | TNFRSF1B_v3 | rs3397 |  |  |  | 0.6393 |
| Chr1:25293084 | RUNX3 region | RUNX3 | rs7536201 |  |  |  | 0.5437 |
| Chr1:67600686 | IL23R region | IL23R_v1 | rs12044149 |  |  |  | 0.2526 |
| Chr1:67611613 | IL23R | IL23R_v2 | rs4655683 |  |  |  | 0.3397 |
| Chr1:67658723 | IL23R | IL23R_v8 | rs12567033 |  |  |  | 0.01 |
| Chr1:67658803 | IL23R | IL23R_v3 | rs72676067 |  |  |  | 0.2915 |
| Chr1:67658954 | IL23R | IL23R_v10 | rs72676069 |  |  |  | 0.3016 |
| Chr1:67670133 | IL23R | IL23R_v11 | rs1004820 |  |  |  | 0.3989 |
| Chr1:67670213 | IL23R | IL23R_v4 | rs1004819 |  |  |  | 0.2987 |
| Chr1:67694202 | IL23R | IL23R_v5 | rs2201841 |  |  |  | 0.3059 |
| Chr1:67705958 | IL23R | IL23R_v7 | rs11209026 |  |  |  | 0.0656 |
| Chr2:204738725 | CTLA4 | CTLA4_v1 | rs231721 |  |  |  | 0.0083 |
| Chr2:204738919 | CTLA4 | CTLA4_v2 | rs3087243 |  |  |  | 0.4366 |
| Chr2:204738938 | CTLA4 region | CTLA4 region | rs11571319 |  |  |  | 0.1652 |
| Chr3:101575882 | NFKBIZ | NFKBIZ | rs595788 |  |  |  | 0.2838 |
| Chr3:101615625 | NFKBIZ region | NFKBIZ_v5 | rs9818678 |  |  |  | 0.4951 |
| Chr3:101615826 | NFKBIZ region | NFKBIZ_v2 | rs4683946 |  |  |  | 0.1998 |
| Chr3:101663317 | NFKBIZ region | NFKBIZ_v7 | rs9881690 |  |  |  | 0.3677 |
| Chr3:101663386 | NFKBIZ region | NFKBIZ_v9 | rs7625614 |  |  |  | 0.4198 |
| Chr3:101663555 | NFKBIZ region | NFKBIZ_v3 | rs7637230 |  |  |  | 0.2087 |
| Chr3:124811314 | SLC12A8 | SLC12A8 | rs651630 |  |  |  | 0.4786 |
| Chr5:150467130 | TNIP1 | TNIP1_v4 | rs140378407 |  |  |  | 0.0049 |
| Chr5:150467170 | TNIP1 | TNIP1_v5 | rs2233279 |  |  |  | 0.4209 |
| Chr5:150467189 | TNIP1 | TNIP1_v1 | rs2233278 |  |  |  | 0.0507 |
| Chr5:150476004 | TNIP1 region | TNIP1_v2 | rs1024995 |  |  |  | 0.1124 |
| Chr5:150476129 | TNIP1 region | TNIP1_v6 | rs11738559 |  |  |  | 0.1709 |
| Chr5:158742950 | IL12B | IL12B_v1 | rs3212227 |  |  |  | 0.2029 |
| Chr5:158750769 | IL12B | IL12B_v2 | rs3213094 |  |  |  | 0.2048 |
| Chr5:158759900 | IL12B region | IL12B_v3 | rs2546890 |  |  |  | 0.4861 |
| Chr5:159912418 | MIR146A | MIR146A | rs2910164 |  |  |  | 0.7600 |
| Chr6:31543031 | TNF-α region | TNFA_v1 | rs1800629 |  |  |  | 0.1624 |
| Chr6:31543101 | TNF-α region | TNFA_v2 | rs361525 |  |  |  | 0.0529 |
| Chr6:31543758 | TNF-α | TNFA | rs3093661 |  |  |  | 0.0319 |
| Chr6:31543827 | TNF−α | TNFA_v3 | rs1800610 |  |  |  | 0.0869 |
| Chr6:31544189 | TNF-α | TNFA | rs3093662 |  |  |  | 0.0779 |
| Chr6:32814975 | TAP1 | TAP1_v4 | rs1135216 |  |  |  | 0.1500 |
| Chr6:32815025 | TAP1 | TAP1_v5 | rs111511638 |  |  |  | 0.0487 |
| Chr6:52101739 | IL17F | IL17F_v1 | rs56499381 |  |  |  | 0.0473 |
| Chr6:52101844 | IL17F | IL17F_v3 | rs2397084 |  |  |  | 0.0942 |
| Chr6:111577761 | TRAF3IP2 region | TRAF3IP2_v1 | rs71562288 |  |  |  | 0.0917 |
| Chr6:111673714 | TRAF3IP2 region | TRAF3IP2_v2 | rs240993 |  |  |  | 0.7282 |
| Chr6:111913070 | TRAF3IP2 | TRAF3IP2_v6 | rs13190932 |  |  |  | 0.0579 |
| Chr6:111913262 | TRAF3IP2 region | TRAF3IP2_v3 | rs33980500 |  |  |  | 0.0786 |
| Chr6:111922503 | TRAF3IP2 | TRAF3IP2_v7 | rs76228616 |  |  |  | 0.0406 |
| Chr6:111922720 | TRAF3IP2 region | TRAF3IP2_v4 | rs13210247 |  |  |  | 0.0662 |
| Chr6:138196066 | TNFAIP3 | TNFAIP3_v1 | rs2230926 |  |  |  | 0.0348 |
| Chr6:138197824 | TNFAIP3 | TNFAIP3_v2 | rs582757 |  |  |  | 0.7371 |
| Chr6:138199417 | TNFAIP3 | TNFAIP3_v3 | rs610604 |  |  |  | 0.6745 |
| Chr9:117552885 | TNFSF15 | TNFSF15_v1 | rs3810936 |  |  |  | 0.6912 |
| Chr9:117558703 | TNFSF15 | TNFSF15_v2 | rs6478108 |  |  |  | 0.6693 |
| Chr9:117566440 | TNFSF15 | TNFSF15_v3 | rs4263839 |  |  |  | 0.6850 |
| Chr9:117568766 | TNFSF15 region | TNFSF15_v4 | rs6478109 |  |  |  | 0.6794 |
| Chr12:6450945 | TNFRSF1A | TNFRSF1A | rs767455 |  |  |  | 0.4326 |
| Chr16:11365416 | SOCS1 region | SOCS1_v2 | rs431918 |  |  |  | 0.1712 |
| Chr16:30942625 | FBXL19 | FBXL19_v3 | rs10782001 |  |  |  | 0.3643 |
| Chr17:26106675 | NOS2 | NOS2_v1 | rs4795067 |  |  |  | 0.3519 |
| Chr17:26124699 | NOS2 | NOS2_v3 | rs16949 |  |  |  | 0.2221 |
| Chr17:26124908 | NOS2 | NOS2_v2 | rs28998802 |  |  |  | 0.1520 |
| Chr21:36470574 | RUNX1 region | RUNX1_v2 | rs2834760 |  |  |  | 0.2097 |
| Chr22:17564907 | IL17RA region | IL17RA_v3 | rs4819553 |  |  |  | 0.1882 |
| Chr22:17565013 | IL17RA region | IL17RA_v4 | rs4819958 |  |  |  | 0.1957 |
